# Odor Impression Prediction from Mass Spectra

**DOI:** 10.1371/journal.pone.0157030

**Published:** 2016-06-21

**Authors:** Yuji Nozaki, Takamichi Nakamoto

**Affiliations:** 1 Department of Computational Intelligence and Systems Science, Tokyo Institute of Technology, Kanagawa, Japan; 2 Precision and Intelligence Laboratory, Tokyo Institute of Technology, Kanagawa, Japan; Francis Crick Institute, UNITED KINGDOM

## Abstract

The sense of smell arises from the perception of odors from chemicals. However, the relationship between the impression of odor and the numerous physicochemical parameters has yet to be understood owing to its complexity. As such, there is no established general method for predicting the impression of odor of a chemical only from its physicochemical properties. In this study, we designed a novel predictive model based on an artificial neural network with a deep structure for predicting odor impression utilizing the mass spectra of chemicals, and we conducted a series of computational analyses to evaluate its performance. Feature vectors extracted from the original high-dimensional space using two autoencoders equipped with both input and output layers in the model are used to build a mapping function from the feature space of mass spectra to the feature space of sensory data. The results of predictions obtained by the proposed new method have notable accuracy (R≅0.76) in comparison with a conventional method (R≅0.61).

## Introduction

Olfaction, one of the chemical senses of human beings, enables us to understand the surrounding environment by perceiving airborne chemicals. Previous studies have suggested that perceived chemical stimuli are associated with the complex organizational structure of the biological olfactory system [1–2], whose input corresponds to a set of olfactory receptors responding to physicochemical properties of airborne chemicals and whose output corresponds to recognition in the cerebrum.

Human beings generally use the following ways to describe an impression of a perceived odor: (1) the name of a chemical, (2) the name of a representative instance, and (3) verbal descriptors. When we describe the impression of a certain chemical, if the chemical is sufficiently familiar, the first way is used. For example, we say ‘sulfur’, ‘ammonia’, etc. However, if the name of the chemical is unknown to the person, concrete examples such as ‘smells like apples’ or ‘smells like rotten eggs’ are often used. The third way may include the prior two methods. That is, when no suitable concrete example can be used to describe the odor, a combination of verbal descriptors of common everyday words, such as ‘floral’, ‘sweet’, and so on, is instead used. In the simplest case, verbal descriptors could just be ‘pleasant’ or ‘unpleasant’ [3–4].

Sensory evaluation tests for odor have been widely adopted to obtain objective impressions quantified by verbal descriptors. The extraction of odor impression is essential not only in the food and cosmetic industries but also in other industries for consumer product evaluation [5]. Since a human cannot continue a sensory test without cessation owing to adaption, conducting sensory evaluation tests to cover a large number of chemicals requires a significant amount of time and resources, and is impractical. In addition, since a large number of physicochemical parameters are associated with chemicals, this prevents us from obtaining a better understanding of the relationship between odor and chemicals. Thus, the aim of this study is to generate a mathematical model from a limited number of samples to predict the impression of a perceived odor.

The Mass spectrum is one of the representative physicochemical properties of chemical substances. Motivated by earlier studies, which clarified the relationship between the scent of a chemical and its chemical structure [6–8], the properties can be utilized in a predictive model. Although it was also shown that odor perception is affected by context [9–10], this fact is less important in our study since the sensory evaluation tests were performed in a standardized environment.

A huge amount of mass spectrum data are available to construct a model to describe odor impression. Several studies have reported on relationships between the odor characteristic of a chemical and its physicochemical parameters by linear modeling approaches such as principal component analysis (PCA) and non-negative matrix factorization (NMF) [11–14]. These studies showed that some fundamental parameters indeed affect our perception of odor. Although PCA and NMF are well-known methods for predictive modeling, they are not suitable for nonlinear data structures [15]. Therefore, since the biological olfactory system is essentially nonlinear, it is difficult to conclude that these linear modeling techniques are wholly compatible with the system. Artificial neural network modeling, one of the definitive methods of nonlinear modeling, is generally used in a broad range of applications. However, few studies have focused on its use in the field of olfaction. Thus, we propose the use of a nonlinear modeling method for odor characteristic prediction that uses an artificial neural network with a deep structure.

## Experiment

**Fig 1:**
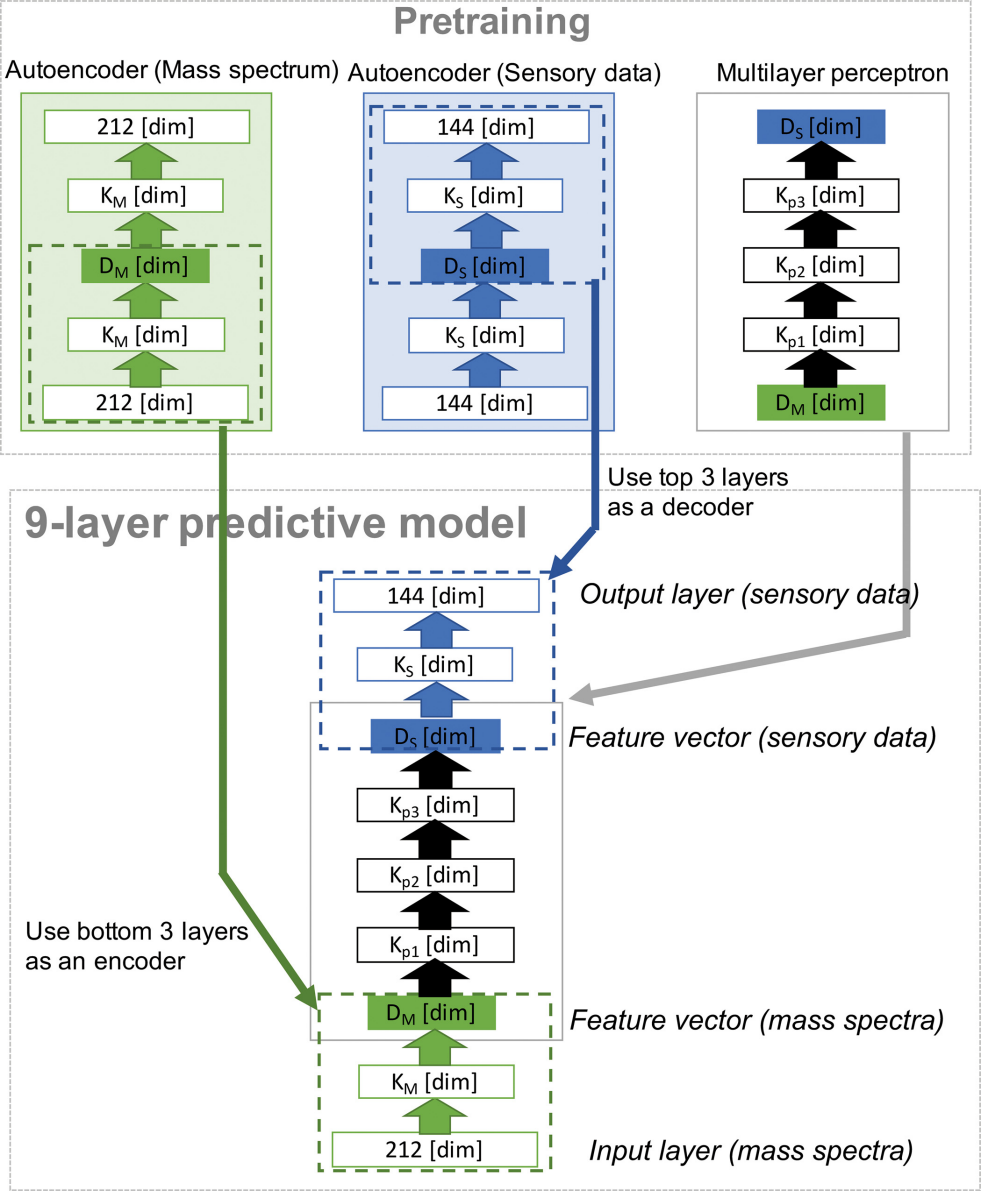
Schematic diagram of the predictive model. The character or number in each box gives the name of the layer or the number of units (dimensionality), respectively.

For the purpose of predicting the odor characteristic of a chemical from its mass spectrum, we designed a predictive model with a nine-layer feed forward neural network. A schematic diagram of the model is shown in Fig 1. Every unit in the layers, except those in the input layer, has a sigmoid function as the activation function.

In general, training a large multilayer neural network has been believed to be difficult for a long time, but studies have shown that such difficulties may be overcome by certain techniques such as feature extraction and weight regularization [16–17]. Accordingly, we trained our model using the following procedure.

First, we calculated the feature vectors separately for mass spectrum data and sensory data. This procedure, called dimensionality reduction, is necessary to avoid *the curse of dimensionality*, the well-known problem which arises when dealing with data having a large number of dimensions [18]. We used an autoencoder with three hidden layers for dimensionality reduction [19]. However, the autoencoders also have a deep structure, making training problematic. We will describe our optimization method in a later section.

Then, the multilayer perceptron located in the middle of the nine-layer predictive model is trained by the feature vectors obtained by the autoencoders mentioned earlier. In other words, this trained multilayer perceptron is a mapping function from the feature space of mass spectra to the feature space of sensory data. After that, we connect the first 3 layers of the autoencoder (which encodes the mass spectrum vector of the original length into the feature vector of the mass spectrum) to the multilayer perceptron for mapping, and the last 3 layers of the autoencoder (which decodes the feature vector of sensory data to the sensory vector of the original length) are also connected to the multilayer perceptron for mapping. Fine-tuning is the final procedure in the modeling [20]. Through this training procedure, the predictive model is able to convert original mass spectrum data into sensory data.

### Data preparation

Two types of dataset are needed to realize the predictive model: the results of a sensory evaluation on monomolecular chemicals and the mass spectra of these chemicals.

The former dataset as above refers to the results of the sensory test previously carried out by Dravnieks, in which 160 odorants were evaluated for each of 146 verbal descriptors on a scale of 0 to 5. The applicability in the test was calculated as the geometric mean of raw scores for ~150 panelists [21]. We used 144 descriptors out of the original 146 (S1 Table). Then for the latter dataset, the mass spectra of chemicals in which the electron ionization method with an energy of 70 [eV] was applied, were obtained from the Chemistry WebBook provided by National Institute of Standards and Technology (http://webbook.nist.gov/chemistry/cas-ser.html). After taking the chemicals common to these two datasets, we obtained 121 chemical samples for the subsequent experiments. Accordingly, the sensory data matrix is expressed as rows for the 121 samples and columns for the 144 descriptors. S2 Table shows a list of the chemicals we used. Intensities with mass-to-charge ratio below 50 might primarily originate from odorless molecules such as oxygen, nitrogen, and carbon dioxide, and intensities with high mass-to-charge ratio originate from molecules with low volatility and have less effect on the odor characteristic. Therefore, we extracted intensities with 51–262 m/z from the original data [22]. Accordingly, the data matrix is expressed by rows for the 121 samples and columns corresponding to 212 intensities. The elements in both matrices were then normalized by dividing by the maximum value in each dataset to obtain a value between 0 and 1.

### Training algorithms

Each sample has hundreds of dimensions, while there are only 100 samples available for the training. When we have a limited number of samples, the predictive capability of the model reduces as the dimensionality increases. Dimensionality reduction, i.e., feature extraction, is a common technique often applied to neural networks to accomplish an effective projection function while avoiding the problems arising from the higher dimensionality.

**Fig 2:**
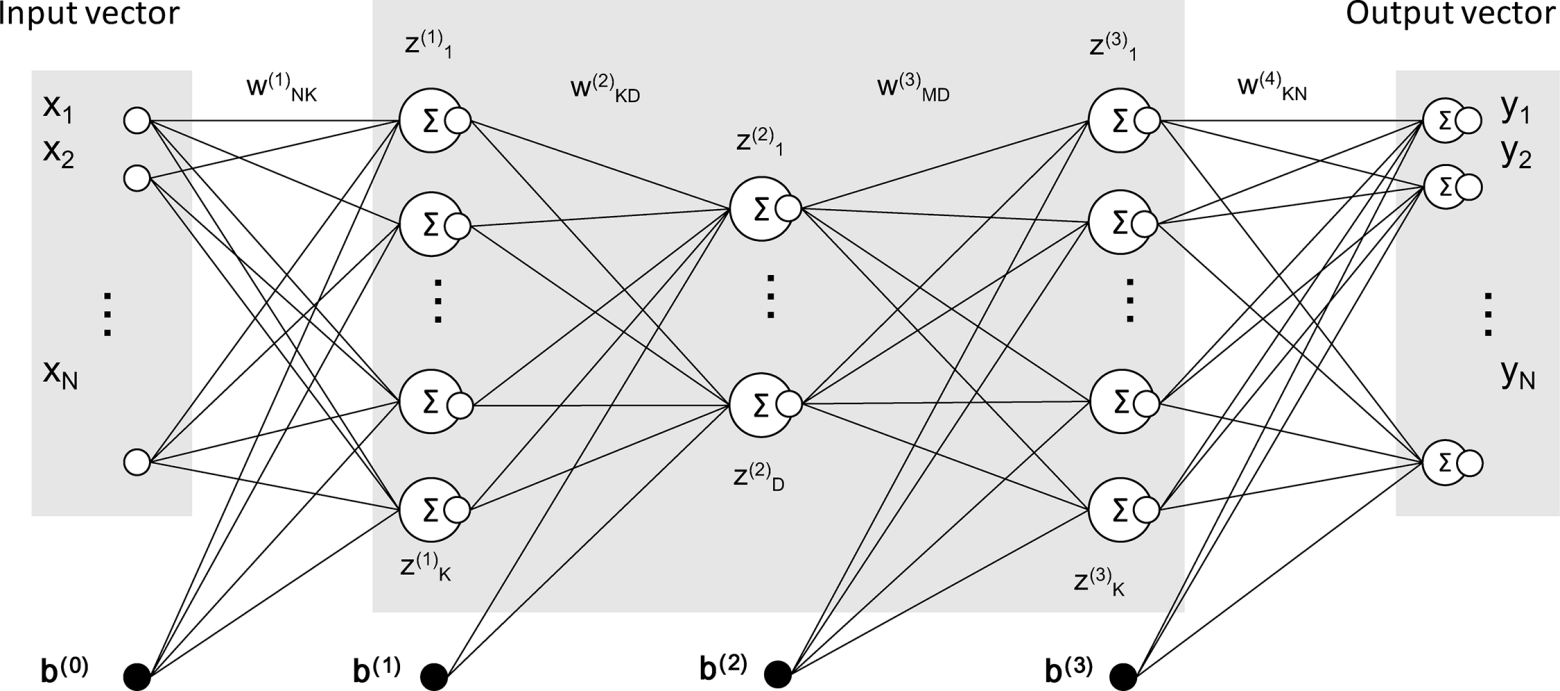
Schematic diagram of an autoencoder. ***x***_***ni***_ corresponds to the *i*th element of the *n*th sample. b^(j)^ is the bias in the *j*th layer. ***y***_***n***_(***x***_***n***_;***W***) = *f*(*W*^(4)^*f*(*W*^(3)^*f*(*W*^(2)^*f*(*W*^(1)^***x***_***n***_ + ***b***^(0)^) + ***b***^(1)^) + ***b***^(2)^) + ***b***^(3)^) is the output of an autoencoder for a given ***x***_***n***_, where ***W*** = {*W*^(4)^,*W*^(3)^,*W*^(2)^,*W*^(1)^,***b***^(0)^,***b***^(1)^,***b***^(2)^,***b***^(3)^}. *f* is a *p*-dimensional sigmoid function, *f*(***a***) = [1/(1 + exp(−*a*_1_)),… 1/(1 + exp(−*a*_*p*_))].

An autoencoder can be regarded as a special family of artificial neural networks, the purpose of which is to learn a compressed representation from a set of data. Fig 2 shows a schematic diagram of an autoencoder. Each autoencoder used in the following experiments consists of an input layer, three hidden layers, and an output layer with the same number of neurons in the input layer. Since fewer neurons are set in the middle hidden layer, a low-dimensional representation can be obtained. A projection function to a low-dimensional representation is acquired through an iterative optimization method known as stochastic gradient descent.

During the optimization process, a training set of *N* vectors {***x***_**1**_,***x***_**2**_,…***x***_***N***_}, which are samples from an original dataset, were used as input vectors. An autoencoder then computes the output ***y***_***n***_(***x***_***n***_;***w***) and updates the parameters to reduce the error function *E*_*n*_(***W***),

$$E_{n}(W)=\overset{M}{\underset{m=1}{\sum }}{\left(y_{nm}-x_{nm}\right)}^{2}+L_{n}(W)$$ (1)

where *L*_*n*_(***W***) is the L1-Norm regularization known as the Lasso [23], which penalizes weights on the basis of the size of the L1 norm of the coefficients,

$$L_{n}(W)=\lambda \underset{l,m,n}{\sum }\left|w_{nm}^{\left(l\right)}\right|$$ (2)

where λ is a positive constant. Accordingly, for any weight w(t)nm(l) in the lth layer in the network including the biases, the update rule is given by

w(t+1)nm(l)=w(t)nm(l)−η∂∂wnm(l)En(W)+α(w(t)nm(l)−w(t−1)nm(l))+noise (3)

where η is the learning rate, which decays with increasing number of iterations, *t* is the number of epochs, and α is the momentum parameter [24–25]. Noise with a normal distribution was added to avoid trapping at a local minimum. A set of weight matrices ***W*** was initialized with random values drawn uniformly from the interval [-0.03, 0.03]. The weights were iteratively updated 200 times for each sample in the training set to reduce the target error function. k-fold cross-validation was applied to prevent overfitting by the predictive model. Both data matrices, the sensory evaluation data and the mass spectra data, were randomly separated into 6 subsets, 5 of which (100 samples) were used for training the whole model and the hold-out set (21 samples) is used for evaluation of the generalization error. We repeat this k-fold cross validation 10 times for different random splittings.

**Fig 3:**
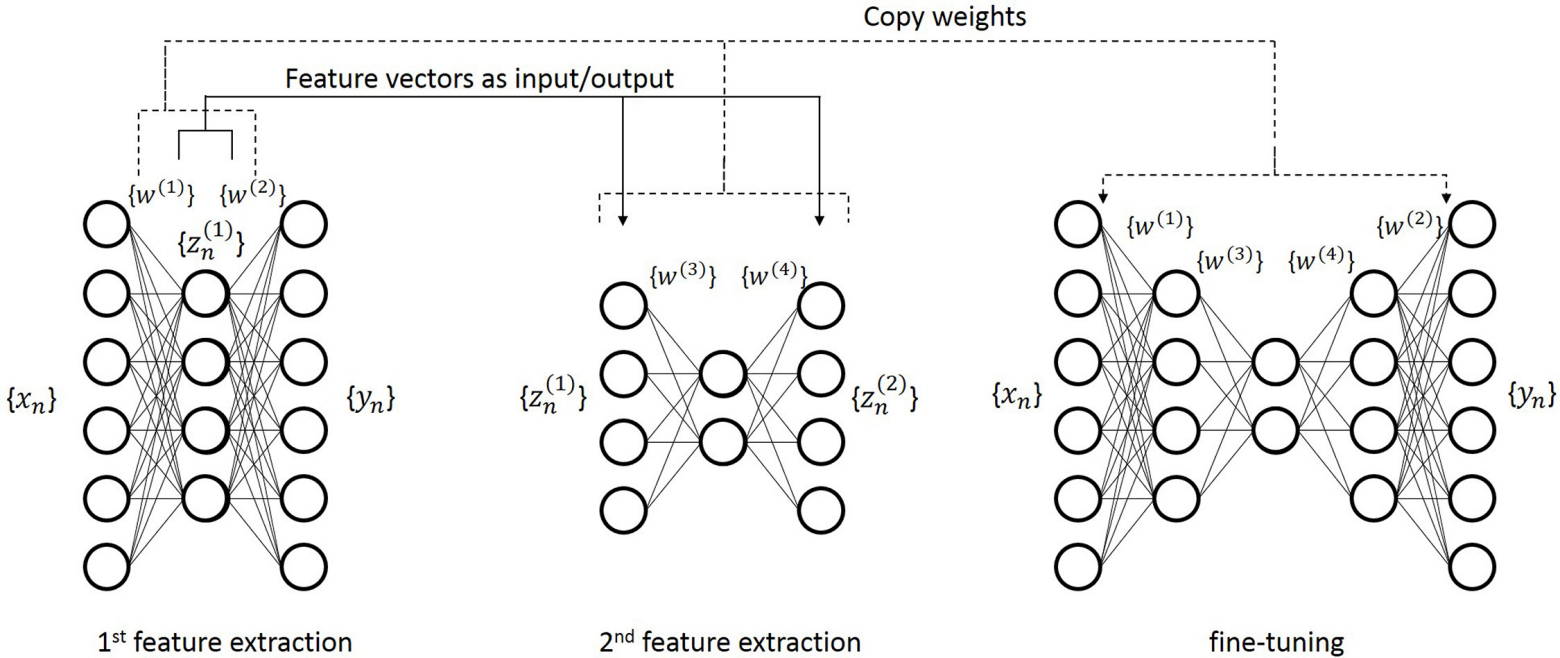
Pretraining procedure for autoencoder. The weights of a 5-layer autoencoder (right) are copied from two 3-layer autoencoders (left and middle).

The generalization error is given as the sum of *E*_*n*_(*w*) calculated for 21 samples in the testing set. It is known that optimizing the parameters and weights in a neural network with multiple hidden layers by the backpropagation algorithm is difficult owing to *the vanishing gradient problem* [26]. Thus, we adopted a pretraining procedure so that the autoencoders give weights that are close to a good solution [27]. Fig 3 shows the procedure. The same update rule and procedure are also applied to the 9-layer predictive model. Then the fine-tuning procedure was performed about 5 times to adjust the entire model to the data.

### Model evaluation

The hold-out set in the 6-fold cross validation, which was excluded from all the training stages was used to evaluate the generalization ability of the models.

Models were repeatedly constructed and evaluated 60 times (6-fold cross validation x 10 times) to obtain the average performance. The number of neurons set in each hidden layer should be optimized by comparison of the sum of reconstruction errors *R*(***W***), where each reconstruction error is calculated by the subtraction of an output vector ***y***_***n***_(***x***_***n***_;***W***) from an input vector ***x***_***n***_:

$$R(W)=\overset{M}{\underset{m=1}{\sum }}\overset{N}{\underset{n=1}{\sum }}\left|x_{nm}-y_{nm}\right|$$ (4)

The prediction capability was evaluated by comparison with the output ***y***_***n***_(***x***_***n***_;***w***) and the original value in the database ***x***_***n***_.

We evaluated the performance of the autoencoders along with that of PCA, commonly used in dimensionality reduction techniques, by comparing reconstruction errors. In addition, the predictive performance of the nine-layer model was compared with that of partial least-squares regression (PLS) [28]. In PLS modeling, the complexity is mainly determined by the number of latent variables used in the model. The same cross-validation rule and evaluation criteria were applied in the selection of the parameter of the model.

Note that neural networks do not always converge, or they are sometimes trapped at a local minimum, providing an anomalous value. Thus, the median of the reconstruction error was used in the evaluation.

## Results

### Performance of autoencoder

To find the optimal number of dimensions for each autoencoder, we iteratively conducted a series of experiments and evaluated the reconstruction error for each model.

**Fig 4:**
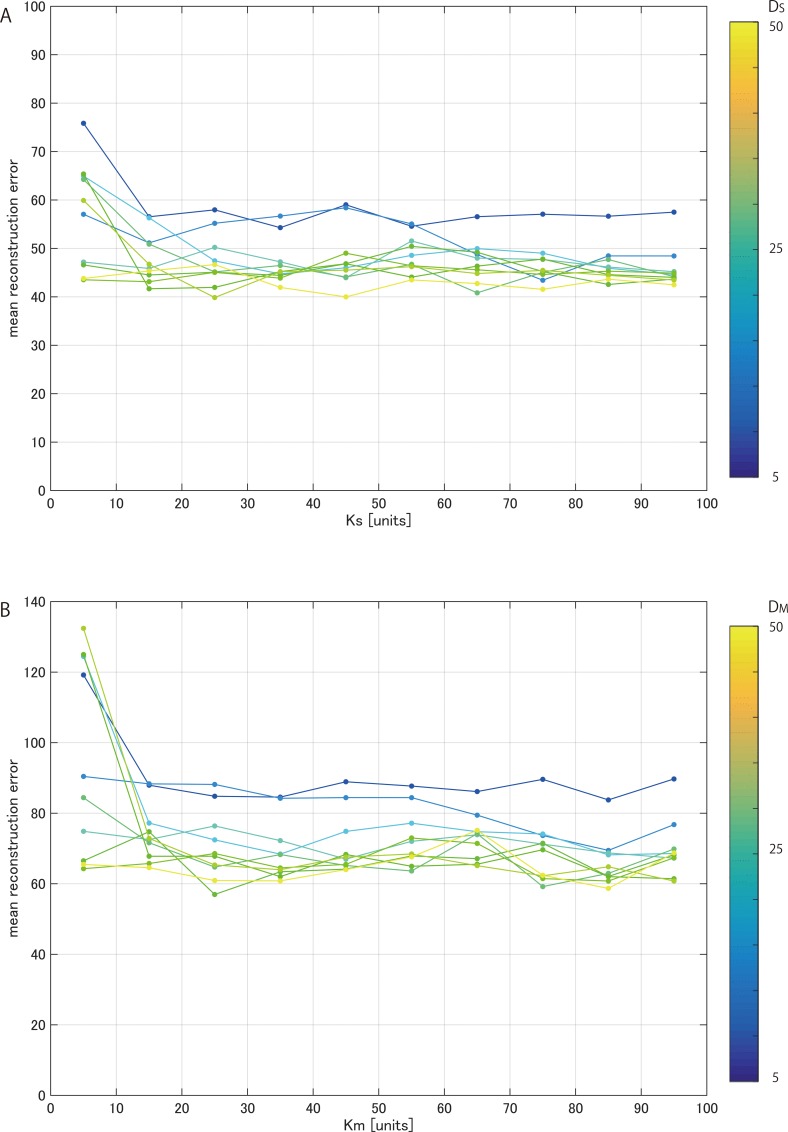
Mean reconstruction errors in cross validation. ***A***, error of autoencoder for sensory data with respect to the number of units K_S_ ***B***, error for mass spectra with respect to the number of units K_M_.

**Fig 5:**
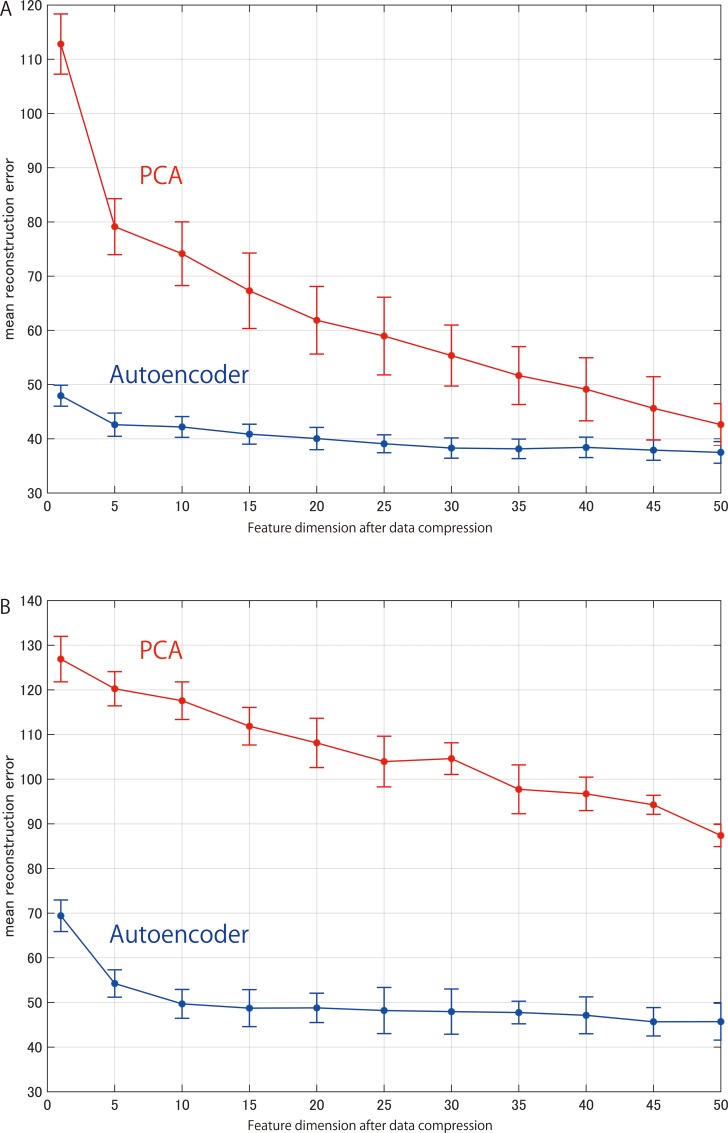
Mean reconstruction errors in cross-validation. Optimal K_S_ (or K_M_), giving the minimum error for each D_S_ (or D_M_) with reference to Fig 4. Error bars indicate standard deviations of testing sample sets. ***A***, error of autoencoder for sensory data with respect to the number of neurons in the hidden layer and error of PCA for sensory data with respect to the number of principal components. ***B***, error for mass spectra with respect to the number of neurons in the hidden layer (in autoencoder) and error with respect to the number of principal components (in PCA).

The number of neurons used in the autoencoder and the number of principal components used in PCA represent the compression efficiency of dimensionality reduction for each model. Figs 4 and 5 show the mean reconstruction errors in 10 repetitions of 6-fold cross validation with respect to the number of neurons in the hidden layers of the autoencoder and the number of principal components of PCA, respectively. The results obtained are summarized in Tables 1 and 2. While each autoencoder has relatively small reconstruction errors, PCA has a larger error at each dimension.

As shown in the Fig 5, the reconstruction errors remained about the same above a certain number of dimensions. To give the predictive model a sufficient margin, 30 dimensions were applied for the autoencoder for the sensory data(D_S_) and 45 dimensions were applied for the autoencoder for the mass spectrum data (D_M_). The reconstruction errors of the autoencoder were much smaller than those of PCA.

**Table 1:** Number of neurons employed in 9-layer predictive model.

	Layer number								
	input	K_M_	D_M_	K_p1_	K_p2_	K_p3_	D_S_	K_S_	output
unit	212	85	45	50	55	50	30	65	144

**Table 2:** Constant coefficients used in updating rule.

Constant Coefficient	Value
η	0.4 × 0.99^(iteration)^
λ	4 × 10^−7^
α	0.025
Noise (normal random)	0.5 × sqrt(2η)

### Performance of predictive model

On the basis of the optimal parameters obtained earlier, we combined 2 autoencoders with a multilayer perceptron and constructed a nine-layer predictive model based on Fig 1. The model was tuned with the same training set used to train the autoencoders.

**Fig 6:**
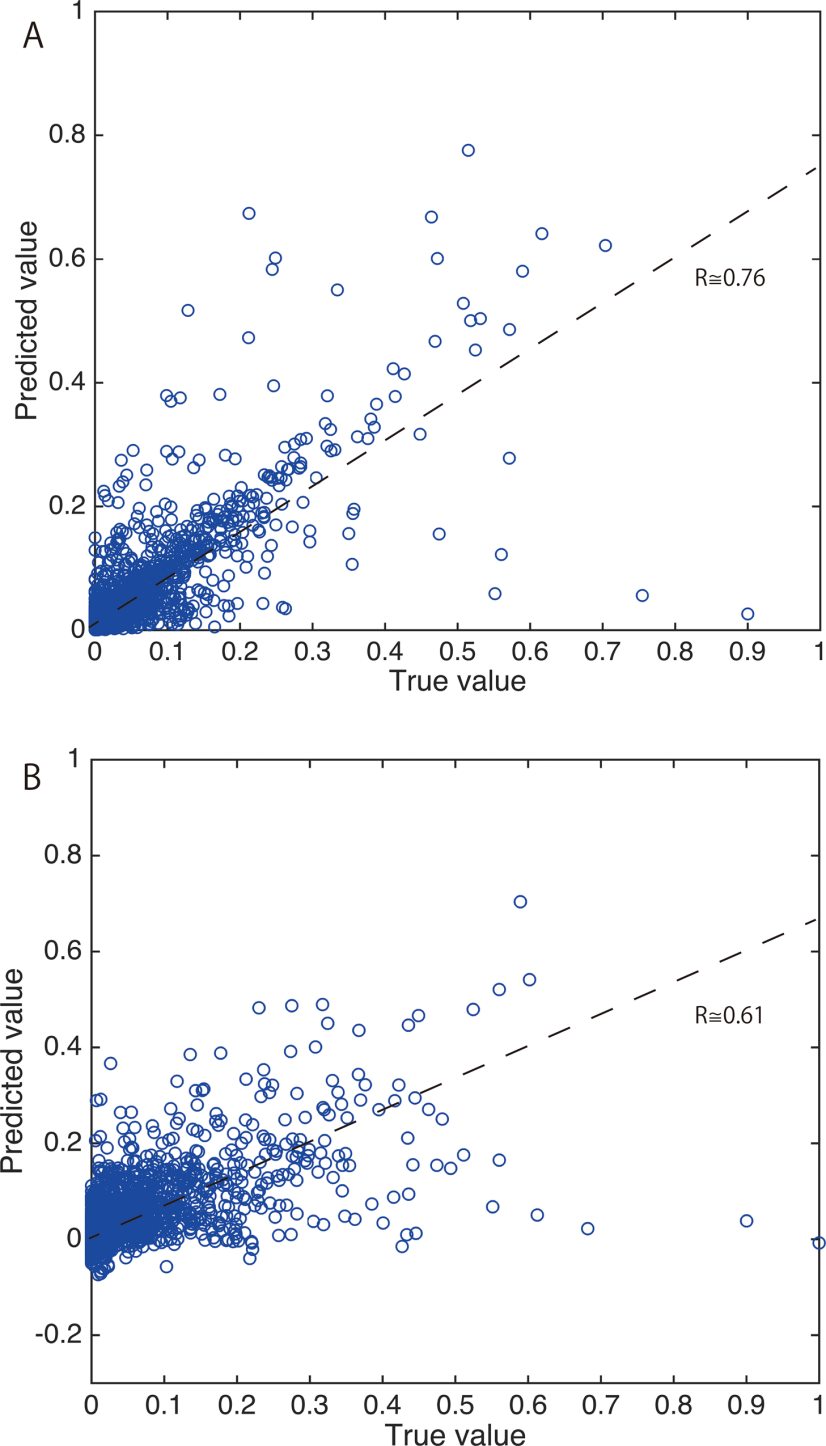
Experimental Result. Examples of predictions by two models, which give a value close to the correlation coefficient for each method. 3024 (= 144 descriptors × 21 samples) data points are plotted in each Fig ***A*,** result for the 9-layer predictive model (R ≅ 0.76), ***B***, result for the PLS model (R ≅ 0.61).

The correlation between predicted values and true values in 10 experiments was then calculated to evaluate the prediction capability of the model in cross-validation. Fig 6 shows examples of the results of prediction by each model. As shown in the figure, the odor character predictions generated by the nine-layer neural model were correlated with human sensory evaluation scores with R ≅ 0.76 in the cross-validation, while the odor character predictions generated by the PLS method using a randomly chosen subset of the dataset resulted in a correlation coefficient about 0.61.

The number of latent variables in the PLS method giving the smallest reconstruction error was found to be 45 for the testing set of 21 samples. Note that PLS is a linear modeling method and does not include any probabilistic factors. Thus, the differences among the 60 PLS models are caused by the sample set used in cross-validation.

### Analysis of the prediction result

**Fig 7:**
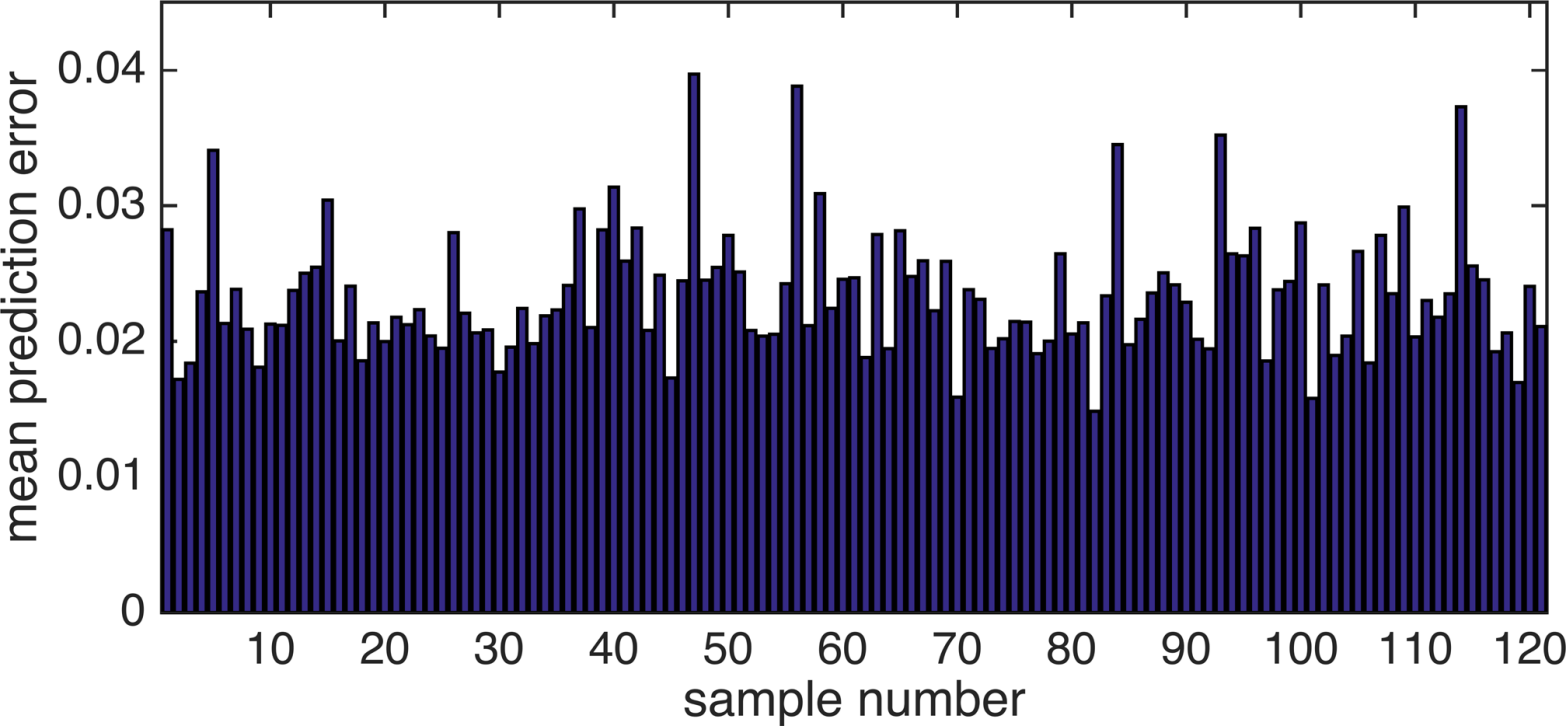
Mean prediction errors of 121 chemical samples. The six most significant six samples (the top 5%) are indicated with the sample number.

**Fig 8:**
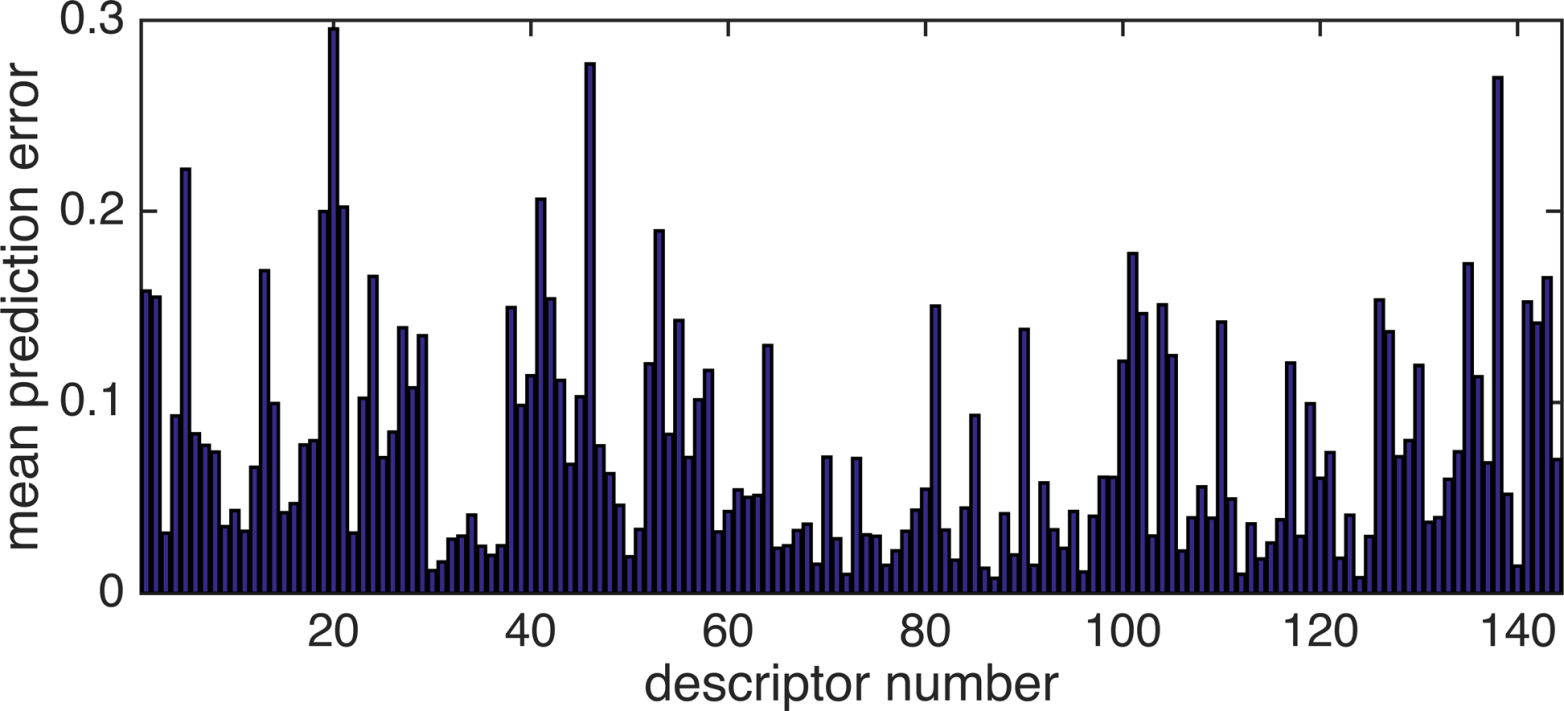
Mean prediction error of dimethylpyrazine (No. 47). The maximum error in the sample was found to have a sensory normalized value of about 0.3.

Analysis of the errors observed in prediction is important to understand the behavior of our model. The mean prediction error of each sample was calculated to obtain the average errors observed in repeatedly performed prediction experiments. Fig 7 shows a bar graph of the mean prediction error for each sample. Dimethylpyrazine (sample number 47) was found to be the sample with the largest error. Fig 8 shows the prediction error of the samples expanded to all 144 descriptors. The maximum error in the sample was found to have a sensory normalized value of about 0.30.

**Fig 9:**
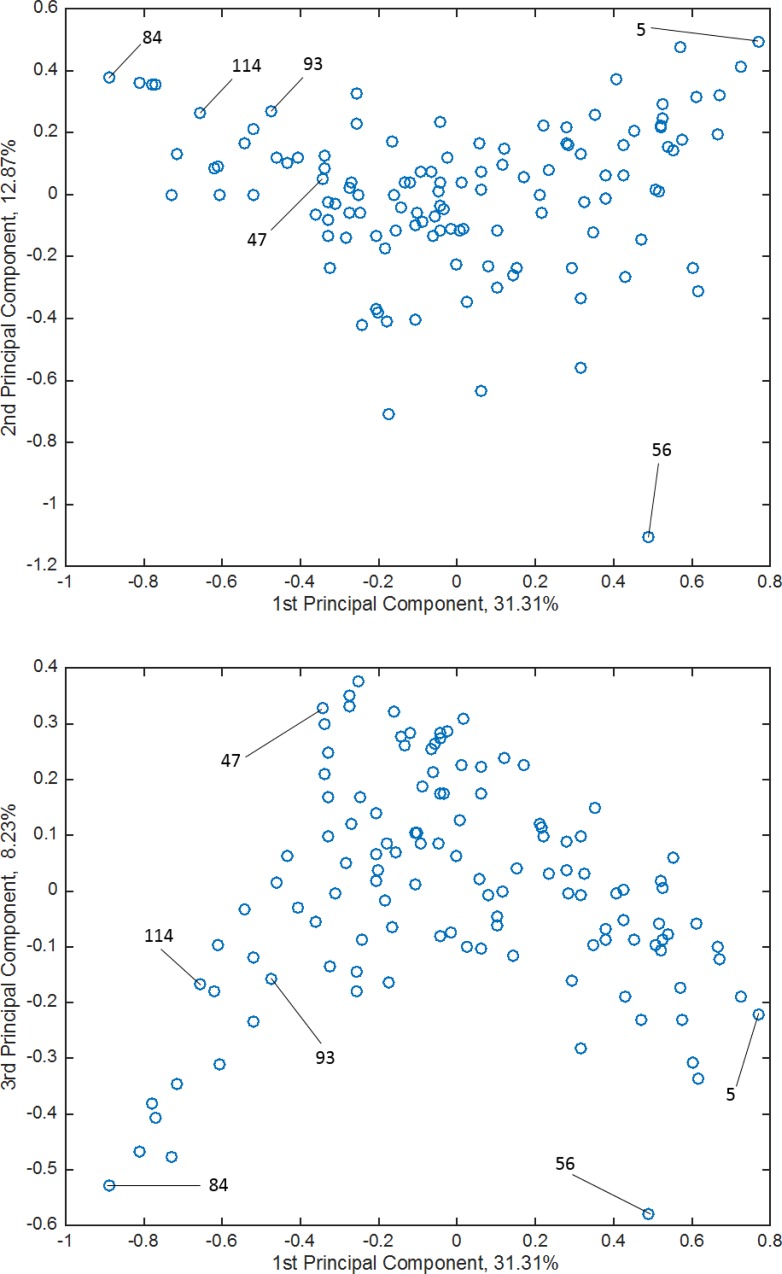
Scatter plots of the result of PCA applied to the original sensory evaluation data. ***A***, first and second principal components. ***B***, first and third principal components.

To further investigate the errors, we chose the most significant six samples (the top 5%) from Fig 7 to find the reason for the large prediction error. On the hypothesis that a particular feature of these six samples prevents them from being predicted precisely, we performed PCA analysis to investigate the distances between the samples in the sensory spaces formed by the first and second principal components and by the first and third principal components. Fig 9 shows the scatter diagrams obtained by applying PCA to the original sensory data. As shown in the figure, the labeled samples are away from the center of the data points in the dependent-variable space and there are few points from which the projection can be learned. It is well known that predicting such data away from the central area with a machine learning method is a very difficult problem. Considering these PCA analyses and the results of comparing two values, the residual of the nonlinear dimensionality reduction and that of the linear dimensionality reduction, we found that the olfactory perceptual space has a nonlinear structure, which was difficult to be captured by a linear modeling method.

## Discussion and Conclusion

In this paper, we proposed a novel method of predicting how humans feel when they smell a chemical by an approach based on machine learning. As a result of our analysis, we obtained the correlation coefficient R ≅ 0.76 in cross validation. The results of this study showed that the odor character of a chemical can be partially predicted from its mass spectrum. Although odor perception is strongly influenced by a posteriori experience, our results support the idea of the dependence of the odor of a chemical on its chemical structure. Although only information from the mass spectrum was used as explanatory variables in our model, accuracy can be improved by utilizing other information on the chemical structure (e.g., molecular weight, functional group).

We also showed the suitability of applying a nonlinear approach towards sensory data on olfaction. By comparing results obtained with predictive models based on linear and nonlinear approaches, we experimentally showed that the relationship between physicochemical properties and olfactory perception is nonlinear. Since a large amount of analysis on sensory data has been carried out using traditional linear methods, new findings can be expected by using nonlinear methods.

## Supporting Information

S1 Table The list of descriptors. (DOCX)

S2 Table The list of odorants and their CAS number. (DOCX)
