# Supplementary material for: Odor Impression Prediction from Mass Spectra
Source: PLoS One. 2016 Jun 21;11(6):e0157030. doi: 10.1371/journal.pone.0157030 (PMC4915715; doi:10.1371/journal.pone.0157030)
Supplement: S1 Table — (DOCX) [file pone.0157030.s001.docx]

S1 Table. The list of descriptors

| Index | Descriptor |
| --- | --- |
| 1 | FRUITY, CITRUS |
| 2 | LEMON |
| 3 | GRAPEFRUIT |
| 4 | ORANGE |
| 5 | FRUITY, OTHER THAN CITRUS |
| 6 | PINEAPPLE |
| 7 | GRAPE JUICE |
| 8 | STRAWBERRY |
| 9 | PEAR |
| 10 | CANTALOUPE, HONEY DEW MELON |
| 11 | PEACH (FRUIT) |
| 12 | BANANA |
| 13 | FLORAL |
| 14 | ROSE |
| 15 | VIOLETS |
| 16 | LAVENDER |
| 17 | COLOGNE |
| 18 | MUSK |
| 19 | PERFUMERY |
| 20 | FRAGRANT |
| 21 | AROMATIC |
| 22 | HONEY |
| 23 | CHERRY (BERRY) |
| 24 | ALMOND |
| 25 | NAIL POLISH REMOVER |
| 26 | NUTTY (WALNUT ETC) |
| 27 | SPICY |
| 28 | CLOVE |
| 29 | CINNAMON |
| 30 | LAUREL LEAVES |
| 31 | TEA LEAVE S |
| 32 | SEASONING (FOR MEAT) |
| 33 | BLACK PEPPER |
| 34 | GREEN PEPPER |
| 35 | DILL |
| 36 | CARAWAY |
| 37 | OAK WOOD, COGNAC |
| 38 | WOODY, RESINOUS |
| 39 | CEDARWOOD |
| 40 | MOTHBALLS |
| 41 | MINTY, PEPPERMINT |
| 42 | CAMPHOR |
| 43 | EUCALIPTUS |
| 44 | CHOCOLATE |
| 45 | VANILLA |
| 46 | SWEET |
| 47 | MAPLE SYRUP |
| 48 | CARAMEL |
| 49 | MALTY |
| 50 | RAISINS |
| 51 | MOLASSES |
| 52 | COCONUT |
| 53 | ANISE (LICORICE) |
| 54 | ALCOHOLIC |
| 55 | ETHERISH, ANAESTHETIC |
| 56 | CLEANING FLUID- |
| 57 | GASOLINE, SOLVENT |
| 58 | TURPENTINE (PINE OIL) |
| 59 | GERANIUM LEAVES |
| 60 | CELERY |
| 61 | FRESH GREEN VEGETABLES |
| 62 | CRUSHED WEEDS |
| 63 | CRUSHED GRASS |
| 64 | HERBAL, GREEN, CUT GRASS |
| 65 | RAW CUCUMBER |
| 66 | HAY |
| 67 | GRAINY (AS GRAIN) |
| 68 | YEASTY |
| 69 | BAKERY (FRESH BREAD) |
| 70 | SOUR MILK |
| 71 | FERMENTED (ROTTEN) FRUIT |
| 72 | BEERY |
| 73 | SOAPY |
| 74 | LEATHER |
| 75 | CARDBOARD |
| 76 | ROPE |
| 77 | WET PAPER |
| 78 | WET WOOL, WET DOG |
| 79 | DIRTY LINEN |
| 80 | STALE |
| 81 | MUSTY, EARTHY, MOLDY |
| 82 | RAW POTATO |
| 83 | MOUSE |
| 84 | MUSHROOM |
| 85 | PEANUT BUTTER |
| 86 | BEANY |
| 87 | EGGY (FRESH EGGS) |
| 88 | BARK, BIRCH BARK |
| 89 | CORK |
| 90 | BURNT, SMOKY |
| 91 | FRESH TOBACCO SMOKE |
| 92 | INCENSE |
| 93 | COFFEE |
| 94 | STALE TOBACCO SMOKE |
| 95 | BURNT PAPER |
| 96 | BURNT MILK |
| 97 | BURNT RUBBER |
| 98 | TAR |
| 99 | CREOSOTE |
| 100 | DISINFECTANT, CARBOLIC |
| 101 | MEDICINAL |
| 102 | CHEMICAL |
| 103 | BITTER |
| 104 | SHARP, PUNGENT, ACID- |
| 105 | SOUR, VINEGAR |
| 106 | SAUERKRAUT |
| 107 | AMMONIA |
| 108 | URINE |
| 109 | CAT URINE |
| 110 | FISHY |
| 111 | KIPPERY (SMOKED FISH) |
| 112 | SEMINAL, SPERM-LIKE |
| 113 | NEW RUBBER • |
| 114 | SOOTY |
| 115 | BURNT CANDLE |
| 116 | KEROSENE |
| 117 | OILY, FATTY |
| 118 | BUTTERY, FRESH BUTTER |
| 119 | PAINT |
| 120 | VARNISH |
| 121 | POPCORN |
| 122 | FRIED CHICKEN |
| 123 | MEATY (COOKED, GOOD) |
| 124 | SOUPY |
| 125 | COOKED VEGETABLES |
| 126 | RANCID |
| 127 | SWEATY |
| 128 | HOUSEHOLD GAS |
| 129 | SULFIDIC |
| 130 | GARLIC, ONION |
| 131 | METALLIC |
| 132 | BLOOD, RAW MEAT |
| 133 | ANIMAL |
| 134 | SEWER |
| 135 | PUTRID. FOUL, DECAYED |
| 136 | FECAL (LIKE MANURE) |
| 137 | CADAVEROUS (DEAD ANIMAL) |
| 138 | SICKENING |
| 139 | DRY, POWDERY |
| 140 | CHALKY |
| 141 | LIGHT |
| 142 | HEAVY |
| 143 | COOL, COOLING |
| 144 | WARM |
