# Supplementary material for: Odor Impression Prediction from Mass Spectra
Source: PLoS One. 2016 Jun 21;11(6):e0157030. doi: 10.1371/journal.pone.0157030 (PMC4915715; doi:10.1371/journal.pone.0157030)
Supplement: S2 Table — (DOCX) [file pone.0157030.s002.docx]

S2 Table. The list of odorants and their CAS number

| Index | CAS Number | Name of Odorant Chemical |
| --- | --- | --- |
| 1 | 698102 | Abhexone |
| 2 | 98862 | Acetophenone |
| 3 | 1122629 | Acetyl Pyridine: ortho-Acetyl Pyridine |
| 4 | 141139 | Adoxal |
| 5 | 77838 | Aldehyde C-16-So-Called |
| 6 | 104610 | Aldehyde C-18-So-Called |
| 7 | 123682 | AUyl Caproate |
| 8 | 123922 | Amyl Acetate: iso-Amyl Acetate |
| 9 | 540181 | Amyl Butyrate |
| 10 | 122407 | Amyl Cinnamic Aldehyde Diethyl |
| 11 | 102192 | Amyl Phenyl Acetate |
| 12 | 2173560 | Amyl Valerate |
| 13 | 104461 | Anethole |
| 14 | 100663 | Anisole |
| 15 | 100527 | Benzaldehyde |
| 16 | 119846 | Benzo Dihydro Pyrone |
| 17 | 5655618 | Bornyl Acetate: iso-Bornyl Acetate |
| 18 | 107926 | Butanoic Acid |
| 19 | 71363 | Butanol: 1-Butanol |
| 20 | 544401 | Butyl Sulfide |
| 21 | 65442311 | Butyl Quinoline: iso-Butyl Quinoline |
| 22 | 76222 | Camphor: dl-Camphor |
| 23 | 99490 | Carvone: 1-Carvone |
| 24 | 87445 | Caryophyllene-beta and gamma Isomers |
| 25 | 33704619 | Cashmeran |
| 26 | 17369594 | Celeriax |
| 27 | 89689 | Chlorothymol |
| 28 | 104552 | Cinnamic Aldehyde |
| 29 | 141275 | Citral |
| 30 | 91645 | Coumarin |
| 31 | 108394 | Cresol: m-Cresol |
| 32 | 106445 | Cresol: p-Cresol |
| 33 | 140396 | Cresyl Acetate: p-Cresyl Acetate |
| 34 | 103935 | Cresyl Butyrate: p-Cresyl-iso-Butyrate |
| 35 | 104938 | Cresyl Methyl Ether: p-Cresyl |
| 36 | 122032 | Cuminic Aldehyde |
| 37 | 1335666 | Cyclocitral: iso-Cyclocitral |
| 38 | 765877 | Cyclohexanedione: 1,2-Cyclohexanedione |
| 39 | 108930 | Cyclohexanol |
| 40 | 80717 | Cyclotene |
| 41 | 25152845 | Decadienal: 2,4-trans-trans-Decadienal |
| 42 | 91178 | Decahydro Naphthalene |
| 43 | 111922 | Dibutyl Amine |
| 44 | 352932 | Diethyl Sulfide |
| 45 | 10094345 | Dimethyl Benzyl Carbinyl Butyrate |
| 46 | 103059 | Dimethyl Phenyl Ethyl Carbinol |
| 47 | 5910894 | Dimethyl Pyrazine: 2,3-Dimethyl Pyrazine |
| 48 | 123320 | Dimethyl Pyrazine: 2,5-Dimethyl Pyrazine |
| 49 | 625843 | Dimethyl Pyrrole: 2,5-Dimethyl Pyrrole |
| 50 | 3658808 | Dimethyl Trisulfide |
| 51 | 4747073 | Diola |
| 52 | 101848 | Diphenyl Oxide |
| 53 | 105544 | Ethyl Butyrate |
| 54 | 105373 | Ethyl Propionate |
| 55 | 13925003 | Ethyl Pyrazine: 2-Ethyl Pyrazine |
| 56 | 470826 | Eucalyptol |
| 57 | 97530 | Eugenol |
| 58 | 6413101 | Fructone |
| 59 | 98011 | Furfural |
| 60 | 98022 | Furfuryl Mercaptan |
| 61 | 111717 | Heptanal |
| 62 | 111706 | Heptanol: 1-Heptanol |
| 63 | 66251 | Hexanal |
| 64 | 142621 | Hexanoic Acid |
| 65 | 111273 | Hexanol: 1-Hexanol |
| 66 | 623370 | Hexanol: 3-Hexanol |
| 67 | 6728263 | Hexenal: trans-2-Hexenal |
| 68 | 111262 | Hexyl Amine |
| 69 | 90879 | Hydratropic Aldehyde Dimethyl Aeetal |
| 70 | 107755 | Hydroxy Citronellal |
| 71 | 120729 | Indole |
| 72 | 14901076 | Ionone: beta-Ionone |
| 73 | 79696 | Irone: alpha-Irone |
| 74 | 138863 | Limonene: d-Limonene |
| 75 | 106729 | Melonal |
| 76 | 2216515 | Menthol: 1-Menthol |
| 77 | 93049 | Methoxy-Naphthalene: 2-Methoxy Naphthalene |
| 78 | 134203 | Methyl Anthranilate |
| 79 | 462953 | Methyl Acetaldehyde Dimethyl Aeetal |
| 80 | 2371428 | Methyl-iso-Borneol: 2-Methyl-iso-Borneol |
| 81 | 491350 | Methyl Quinoline: para-Methyl Quinoline |
| 82 | 2459098 | Methyl iso-Nicotinate |
| 83 | 119368 | Methyl Salicylate |
| 84 | 2432511 | Methyl Thiobutyrate |
| 85 | 1222055 | Musk Galaxolide |
| 86 | 1506021 | Musk Tonalid |
| 87 | 37677148 | Myracaldehyde |
| 88 | 143135 | Nonyl Acetate |
| 89 | 4674504 | Nootkatone |
| 90 | 111875 | Octanol: 1-Octanol |
| 91 | 3391864 | Octenol: l-Octen-3-OL |
| 92 | 109524 | Pentanoic Acid |
| 93 | 591800 | Pentenoic Acid: 4-Pentenoic Acid |
| 94 | 103822 | Phenyl Acetic Acid |
| 95 | 536743 | Phenyl Acetylene |
| 96 | 60128 | Phenyl Ethanol |
| 97 | 78591 | Phorone: iso-Phorone |
| 98 | 80568 | Pinene: alpha-Pinene |
| 99 | 105668 | Propyl Butyrate |
| 100 | 135795 | Propyl Quinoline: iso-Propyl Quinoline |
| 101 | 111477 | Propyl Sulfide |
| 102 | 110861 | Pyridine |
| 103 | 94597 | Safrole |
| 104 | 115719 | Santalol |
| 105 | 83341 | Skatole |
| 106 | 10482561 | Terpineol, mostly alpha-Terpineol |
| 107 | 110010 | Tetrahydro Thiophene |
| 108 | 91612 | Tetraquinone |
| 109 | 123933 | Thioglycolic Acid |
| 110 | 110021 | Thiophene |
| 111 | 89838 | Thymol |
| 112 | 529204 | Tolualdehyde: ortho-Tolualdehyde |
| 113 | 108883 | Toluene |
| 114 | 75503 | Trimethyl Amine |
| 115 | 104676 | Undecalactone: gamma-Unadecalactone |
| 116 | 112389 | Undecylenic Acid |
| 117 | 590863 | Valeraldehyde: iso-Valeraldehyde |
| 118 | 503742 | Valeric Acid: iso-Valeric Acid |
| 119 | 108292 | Valerolactone: gamma-Valerolactone |
| 120 | 121335 | Vanillin |
| 121 | 122485 | Zingerone |
